# Common Denominator of MASLD and Some Non-Communicable Diseases

**DOI:** 10.3390/cimb46070399

**Published:** 2024-06-29

**Authors:** Katarzyna Ferenc, Sara Jarmakiewicz-Czaja, Aneta Sokal-Dembowska, Katarzyna Stasik, Rafał Filip

**Affiliations:** 1 Institute of Medicine, Medical College of Rzeszow University, 35-959 Rzeszow, Poland; kferenc@ur.edu.pl (K.F.);; 2 Institute of Health Sciences, Medical College of Rzeszow University, 35-959 Rzeszow, Poland; sjczaja@ur.edu.pl (S.J.-C.); asokal@ur.edu.pl (A.S.-D.); 3 IBD Unit, Department of Gastroenterology, Clinical Hospital No. 2, 35-301 Rzeszow, Poland

**Keywords:** cardiovascular diseases, diabetes, liver cancer, obesity, steatotic liver disease

## Abstract

Currently, steatohepatitis has been designated as metabolic dysfunction-associated steatohepatitis (MASLD). MASLD risk factors mainly include metabolic disorders but can also include genetic, epigenetic, and environmental factors. Disease entities such as obesity, diabetes, cardiovascular disease, and MASLD share similar pathomechanisms and risk factors. Moreover, a bidirectional relationship is observed between the occurrence of certain chronic diseases and MASLD. These conditions represent a global public health problem that is responsible for poor quality of life and high mortality. It seems that paying holistic attention to these problems will not only help increase the chances of reducing the incidence of these diseases but also assist in the prevention, treatment, and support of patients.

## 1. Introduction

Metabolic dysfunction-associated steatohepatitis (MASLD) is the latest definition and evidence of interest in the development and treatment of steatohepatitis, which is associated with the metabolic syndrome. Undoubtedly, the incidence of MASLD has been increasing recently. In turn, the prevalence of MASLD is strongly correlated with the incidence of obesity, diabetes, cardiovascular disease (CVD), and liver cancer, among others. Between these disease entities, there are strong common risk factors and biochemical mechanisms, as well as similarities in the direction of their development and complications [1].

Obesity is a growing problem for the health care systems of many countries. It is a disease characterized by excessive fat accumulation and abnormal fat distribution, with negative effects on health [2,3]. The causes of obesity are multifactorial and bidirectional and include a predisposition to many diseases, such as type 2 diabetes, high blood pressure, lipid disorders, and many others [4,5,6]. According to the World Health Organization (WHO), in 2019, type 2 diabetes (T2DM) directly caused 1.5 million deaths, with 460,000 deaths attributed to renal complications. Additionally, elevated glucose levels are believed to be the cause of 20% of deaths from cardiovascular issues [7]. Both in developed and developing nations, there is a continuous rise in the prevalence of chronic conditions, such as MASLD and T2DM. It is estimated that MASLD affects roughly 70–80% of individuals with diabetes [8]. Furthermore, correlations between diabetes, cardiovascular disease, and MASLD show that the risk of death in these patients increases as the severity of liver fibrosis increases [9,10]. In addition, end-stage liver disease, along with all of the above contributing factors, can lead to the development of hepatocellular carcinoma (HCC), which is among the most common cancers worldwide [10,11,12]. Therefore, it is important and vital to understand the mechanisms of MASLD and its relationship to other diseases, not only to detect it early but also to assess its risk. MASLD is a heterogeneous disease. Risk factors include gender, age, ethnicity, metabolic status, and genetic predisposition [8]. In addition, the pathogenesis of MASLD has been attributed to lipotoxicity, mitohondrial dysfunction, lysosome dysfunction, intestinal dysbiosis, or ferroptosis. Currently, drug treatment is under investigation. It is lifestyle changes that are of major importance in the prevention and treatment of MASLD and any comorbidities [10]. Therefore, the purpose of this review is to highlight the role of common mechanisms linking MASLD with obesity, T2DM, CVD, and liver cancer.

## 2. Obesity and Metabolic Dysfunction-Associated Steatotic Liver Disease

Quek et al. estimate that the incidence of MASLD has increased significantly over the past 20 years. Furthermore, the incidence of the disease has increased in parallel with an increase in the prevalence of obesity. It has been shown that 75% of people with obesity may have MASLD [13]. Therefore, one of the diagnostic criteria for MASLD is the presence of overweight or obesity and the presence of one of the metabolic risk abnormalities, which include a waist circumference ≥102/88 cm in Caucasian men and women (or ≥90/80 cm in Asian men and women) [14].

One of the factors predisposing to MASLD in obese patients is genetic [15,16,17,18,19,20]. Another is lifestyle, including diet and a lack of physical activity.

### 2.1. Lifestyle

Another component of the link between obesity and MASLD is lifestyle, which consists of improper eating habits such as a diet high in fat, mainly saturated fatty acids and trans-isomeric fatty acids, fructose, and a lack of or low levels of physical activity.

#### 2.1.1. High-Fat Diet

High-fat diets, in which fat accounts for 45–75% of daily energy needs, have been shown to have an association with the occurrence of hepatic steatosis [21]. In their study on animal models, which included a diet high in 60% fat lasting 80 weeks, Velázquez et al. observed an increase in body weight in mice and hepatic steatosis with portal inflammation and progressive organ fibrosis. In addition, the authors of the study point to the presence of endoplasmic reticulum (ER) stress associated with obesity, which can lead to organ steatosis by altering lipid metabolism in the liver [22]. Dobbie et al. studied the effect of a low-calorie diet (LCD) on the efficacy of treatment for people with MASLD and comorbid obesity. The LCD used was characterized by three variations: an intake of 800–1500 kcal/day, a caloric reduction of 500 kcal/day, or a caloric reduction of 25% of daily energy requirements. The researchers showed that this type of diet reduced the body weight of obese patients, as well as the liver fat content, and also improved serum transaminase values [23]. In another study, De Nucci et al. used a very low-calorie ketogenic diet (VLCKD), which was carried out for 8 weeks. These were adult overweight or obese patients, in whom the amount of liver fat was also examined. They observed no reduction in inflammation and a reduction in the degree of organ steatosis [24]. However, it should be noted that it is primarily the combination of excess fat and carbohydrates in the diet that is wrong.

#### 2.1.2. A Diet High in Fructose

The intestinal catabolism of fructose may have a protective effect on the liver, while overexposure and its metabolism in the liver may predispose to MASLD [25]. In a study by Shapiro et al., the authors observed that chronic intake of significant amounts of fructose can predispose to resistance to leptin, with an indirect effect on weight gain, which in turn can induce the cascade of liver steatosis [26]. Conversely, removing fructose from the diet can reverse leptin resistance [27]. Excess fructose consumption can also lead to the induction of excessive oxidative stress, inflammation, or even liver fibrosis [28]. Fructose is an inducer of de novo lipogenesis (DNL). Through DNL, fructose can contribute to increased oxidative stress, which then contributes to inflammation. Furthermore, visceral fat mass may increase with a high fructose diet, so it may indirectly alter the profile of cytokines and adipokines [29]. In addition, intermediate fructose metabolites may act as regulators of certain transcription factors that control lipogenesis or gluconeogenesis [30]. Fructose also affects molecular determinants of liver steatosis (animal model studies), e.g., fatty acid synthase (Fasn), acetyl-coenzyme carboxylase (Acaca), L-type pyruvate kinase (Pklr), and transcription factor binding sterol regulatory element 1 (Srebp1c) [31].

#### 2.1.3. Sedentary Lifestyle

Low or no physical activity predisposes to excessive body weight, which, in turn, predisposes to MASLD. Romero-Gómez et al. indicate that different types of exercise (aerobic, resistance, and others) show similar effects on liver fat burning (about 20–30%). However, by introducing physical activity, weight reduction is more effective, and this in turn reduces liver fat (up to 80%) [32]. Patients are recommended to introduce more than 150 min per week of moderate-intensity aerobic training in 3–5 sessions [33]. Physical activity and dietary interventions are considered effective therapies for MASLD, and they can upregulate lipophagy in the liver. Due to different molecular pathways, a combination of two therapies (diet and physical activity) can intensify the effectiveness of treatment [34]. In addition, exercise is associated with a reduced risk of advanced liver fibrosis, associated with MASLD [35]. In addition to reducing organ steatosis, the introduction of physical activity can also reduce the levels of inflammatory markers and visceral adipose tissue [36].

### 2.2. Gut Microbiota Disorders vs. MASLD

**Figure 1:**
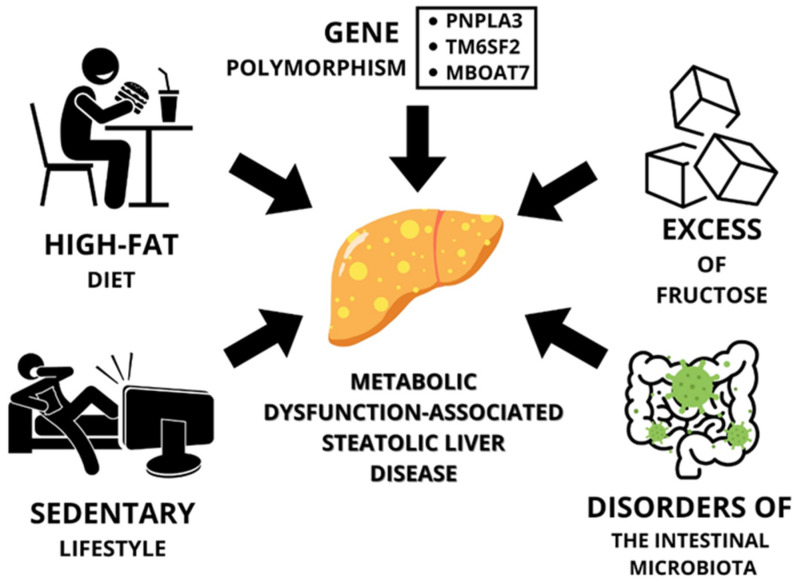
Summary of predisposing factors for obesity-related MASLD.

Another factor predisposing to MASLD in obesity is the disruption of the gut microbiota, but this is strongly associated with a poor diet and a sedentary lifestyle. Liver steatosis may be associated with a change in the gut microbiota, both quantitatively and qualitatively. An increase in *Proteobacteria* and *Firmicutes* is observed, while a decrease in *Bacteroidetes* is observed [37]. In addition, at each stage of organ steatosis and at later stages, the composition of the gut microbiota changed [38]. Furthermore, Tilg et al. concluded in their paper that intestinal barrier dysfunction, which can affect the translocation of pathogenic bacteria or their metabolites, affects intestinal–liver communication. Endotoxin, which can enhance the release of pro-inflammatory cytokines, may be associated with low-grade inflammation. Endotoxemia shows an association with the severity of liver disease [39]. In addition to endotoxins, peptidoglycans and bacterial DNA can also accelerate the development of liver disease [40]. Therefore, intestinal dysbiosis can promote the progression of liver steatosis to the next stages, such as inflammation or organ fibrosis [41]. Leung et al., in their article, concluded that intestinal dysbiosis may be one of the main factors associated with the heterogeneous phenotype of MAFLD (metabolic-associated fatty liver disease) [42]. In contrast, Hu et al. pointed out that it would be appropriate to consider and direct research to viruses, fungi, or intestinal archaeons, not just intestinal bacteria, in the context of the link to liver disease [43]. In turn, Lang et al. stated that the therapeutic approach should be individualized with respect to the introduction of specific bacterial strains to intensify drug therapy [44]. A summary of obesity-related predisposing factors for MASLD is shown in Figure 1.

## 3. Type 2 Diabetes and Metabolic Dysfunction-Associated Steatotic Liver Disease

Available data suggest a strong link between the presence of insulin resistance (IR) and MASLD, affecting both obese and non-obese populations [45]. Research by Jäger et al. has shown a positive correlation between the fatty liver index (FLI) and the risk of type 2 diabetes in both genders, with FLI values associated with markers of dyslipidemia, liver enzymes, fetuin A, and CRP levels [46]. While both MASLD and T2DM are less common in women compared to men, MASLD can contribute to the development of T2DM regardless of gender [47]. Studies by Muzic et al. highlighted a bidirectional relationship between MASLD and T2DM, where MASLD can lead to insulin resistance and an increased risk of T2DM, while T2DM may worsen the progression to MASLD [48]. The presence of MASLD is considered a predictor of the risk of type 2 diabetes [47]. Zeng et al. have shown a positive association between MASLD and increased insulin resistance markers, such as insulin resistance index (HOMA-IR) and TyG-BMI (triglyceride-glucose index with body mass index), within the non-diabetic population [45]. Conversely, improving MASLD can reduce the future risk of T2DM, emphasizing the importance of implementing lifestyle changes and dietary interventions to mitigate the severity of fatty liver disease [47].

### 3.1. High Supply of Fats and Carbohydrates in the Diet

**Figure 2:**
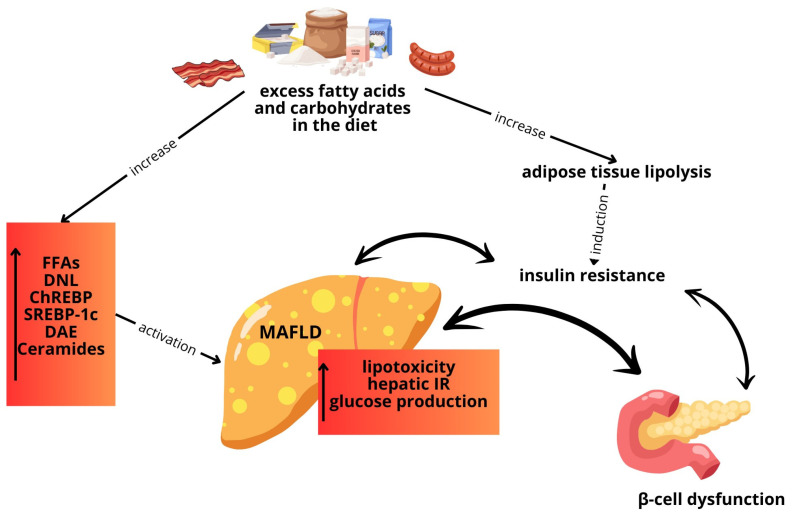
Abnormal hepatic lipid profiles and decreased hepatic triglyceride (TG) secretion lead to fat accumulation, insulin resistance, and β-cell dysfunction, increasing the risk of type 2 diabetes. MAFLD: metabolic dysfunction-associated fatty liver disease; FFAs: free fatty acids; TG: triglyceride; ChREBP: carbohydrate response element-binding protein; SREBP1c: sterol regulating element-binding protein 1c; DNL: de novo lipogenesis; DAE: diacylglycerols.

There are at least two mechanisms that may contribute to the development of insulin resistance and MASLD. One of these mechanisms is closely tied to the dietary intake of fats and carbohydrates. High consumption of dietary fats can lead to elevated levels of free fatty acids, increased *de novo lipogenesis* (DNL), and consequently reduced secretion of triglycerides (TGs) from the liver. Similarly, excessive intake of carbohydrates can stimulate the DNL pathway, ultimately leading to the onset of fatty liver disease (Figure 2) [49].

Within the DNL pathway, certain lipogenic molecules like acetyl-CoA carboxylase (ACC), stearoyl CoA-desaturase-1 (SCD-1), and fatty acid synthesis (FAS) are produced [49]. Insulin and glucose primarily regulate the DNL pathway, with glucose activating DNL via the transcription factor carbohydrate response element-binding protein (ChREBP) and insulin promoting DNL by upregulating sterol regulating element-binding protein 1c (SREBP-1c), the master transcription factor for lipogenesis [50]. SREBP-1c is implicated in the generation of diacylglycerol (DAG) and ceramides, molecules that induce inflammation by interacting with tumor necrosis factor-α (TNF-α), thereby exacerbating insulin resistance [49].

**Figure 3:**
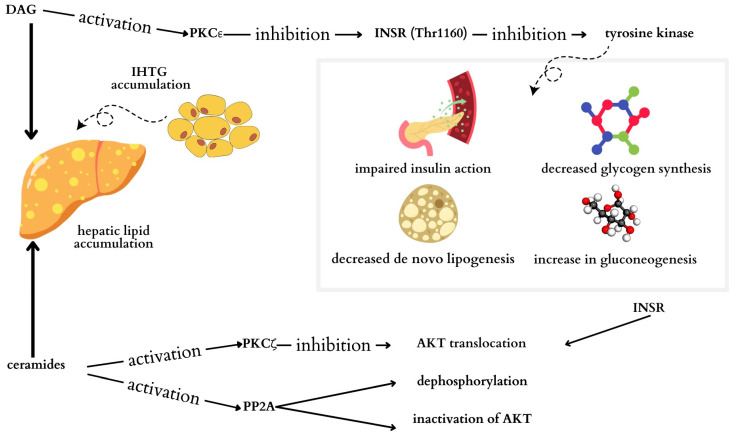
Potential mechanism leading to the development of hepatic insulin resistance mediated by diacylglycerols (DAGs) and ceramides. Increased intrahepatic triglycerides (IHTGs) lead to the accumulation of sn-1,2-diacylglycerol. DAG activates protein kinase C (PKC) isoforms; activation of the ε isoform (PKCε) occurs in the insulin-resistant liver. PKCε phosphorylates the insulin receptor (INSR) Thr1160, leading to inhibition of INSR tyrosine kinase activity. Ceramide activation of protein kinase C-ζ (PKCζ) results in impaired translocation of AKT to the cell membrane, thereby interfering with the involvement of AKT in insulin action. Additionally, ceramide activation of protein phosphatase 2A may lead to dephosphorylation and inactivation of AKT. PKCε: protein kinase Cϵ; INSR: receptor insulin; PKCζ: protein kinase C zeta; AKT: protein kinase B; PP2A: protein phosphatase 2A.

Additionally, two classes of lipids, DAG and ceramide, play crucial roles in the development of insulin resistance (Figure 3) [51]. Studies have shown a direct correlation between total DAG levels and the HOMA-IR [52,53]. In patients with increased intrahepatic triglycerides (IHTGs), sn-1,2-diacylglycerol accumulates, which is responsible for the activation of protein kinase C (PKC) isoforms, especially the ε isoform (PKCε). As a result of phosphorylation of the insulin receptor (INSR) Thr1160 by PKCε, INSR tyrosine kinase is inhibited, and insulin action is impaired [54].

It has been observed that the presence of ceramides in the liver is associated with hepatic IR in humans. Ceramide activation of protein kinase C-ζ (PKCζ) probably impairs the translocation of protein kinase B to the cell membrane, preventing its participation in the action of insulin [54]. Insulin signaling may also be dependent on protein phosphatase 2A through the inactivation of certain kinases [55]. Ceramide activation of protein phosphatase 2A may lead to dephosphorylation and inactivation of protein kinase B [54].

In a state of insulin resistance, the liver is unable to suppress hepatic glucose production in response to insulin, thus leading to the severity of DNL [56]. The inability to inhibit glucose production in the liver and promote lipid synthesis leads to the development of hyperglycemia and hypertriglyceridemia [57]. Increased hepatic gluconeogenesis is likely the main cause of fasting hyperglycemia in T2DM. In turn, increased lipid accumulation in the liver and skeletal muscles may impair insulin signaling and cause insulin resistance. Additionally, overexpression of lipoprotein lipase in the liver and skeletal muscles may induce peripheral insulin resistance [58].

In the second mechanism, attention is drawn to oxidative stress, inflammatory mediators, lipid peroxidation, and mitochondrial dysfunction [49]. IR is associated with chronic low-grade inflammation seen in patients with diabetes and MASLD [59]. Pro-inflammatory cytokines and transcription factors are highly expressed, among others, in the liver and adipose tissue [60]. It has been observed that some pro-inflammatory cytokines, such as TNF-α and interleukin-6 (Il-6), can activate nuclear factor-κB kinase (IKK), complex IKKβ, and c-Jun N-terminal kinase (JNK). Additionally, Il-6 induces suppressors of cytokine signaling (SOCS) 1 and 3, which may result in impaired insulin signaling and the development of IR. Moreover, IKKβ and JNK can activate nuclear factor kappa-B (NF-κB) and cause its translocation to the nucleus, and endoplasmic reticulum (ER) stress can additionally activate the JNK pathway [61].

Presumably immune-related inflammatory changes, insufficient inhibition of lipolysis, and excess FFA cause ectopic lipid deposition, and pro-inflammatory factors secreted by adipose tissue, such as TNF-α, IL6, and IL-1β, may impair insulin signaling [58,61].

### 3.2. Genetic Factors

Dongiovanni et al. demonstrated that hepatic steatosis is causally related to insulin resistance in patients at risk of metabolic dysfunction-associated steatohepatitis (MASLD). The authors indicated that some genetic variants, TM6SF2 E167K (rs58542926) and GCKR P446L (rs1260326), increase liver fat content and may be associated with a moderate increase in insulin resistance and the risk of T2DM [62].

## 4. Cardiovascular Disease and Metabolic-Related Steatohepatitis

Hepatic steatosis is an independent risk factor for the development of cardiovascular disease [11]. In addition, the risk increases when patients have comorbidities such as T2DM or hypertriglyceridemia [11,63]. The pathophysiological mechanisms responsible for MASLD are considered to be an altered lipid profile, insulin resistance, endothelial dysfunction, and chronic inflammation [63]. A 2021 meta-analysis showed that MASLD is associated with a moderately increased risk of fatal or non-fatal CVD events [64]. In contrast, a population-based evaluation in the Framingham study showed that hepatic steatosis is associated with coronary artery and abdominal aortic calcification, regardless of the presence of other conditions that increase cardiovascular risk [65].

### 4.1. Risk Factors

#### 4.1.1. Chronic Inflammation

MASLD is associated with increased production of pro-inflammatory cytokines and increased levels of serum liver enzymes. Patients with MASLD have an increase in the production of CRP protein, oxidized LDL, and plasminogen activator inhibitor-1, as well as other inflammatory proteins mediated by IL-6 and TNF-α These factors in patients with MASLD are associated with an increased risk of CVD and vascular atherosclerosis [66,67].

#### 4.1.2. Insulin Resistance

MASLD is associated with peripheral and hepatic insulin resistance. Disorders in the course of tissue insulin resistance and an increase in peripheral and hepatic insulin secretion also lead to atherogenic dyslipidemia and the release of pro-inflammatory factors, vasoactive factors, and thrombogenic factors, which promote the development of hypertension and cardiovascular disease [68]. Insulin resistance increases de novo lipogenesis in MASLD. In the course of insulin resistance, there is a persistence of high levels of insulin in the blood, which leads to an abnormal mechanism. It is characterized by an increase in circulating blood levels of glucose and free fatty acids, stimulating the release of triacylglycerol from hepatocytes, which can lead to the development of atherosclerotic disease [69]. High glucose levels induce oxidative stress and contribute to the inflammatory response, leading to cellular damage. In addition, hyperglycemia affects coronary artery disease by increasing endothelial monocyte adhesion, enhancing vascular smooth muscle cell proliferation, and indirectly causing endothelial dysfunction [68]. MASLD patients also experience increased platelet activity, elevated levels of clotting factors, and fibrinolysis inhibitors, which may contribute to the development of atherosclerotic thrombosis [70,71].

#### 4.1.3. Abnormal Lipid Profile

Patients with MASLD exhibit a proatherogenic lipid profile characterized by high triglycerides, low-density lipoprotein cholesterol (LDL-C), reduced high-density lipoprotein (HDL-C) levels, and high apolipoprotein B (apoB) levels [72]. Studies indicate that plasma apoB concentration is the strongest predictor of CVD risk [68]. Another mechanism explaining the increase in CVD risk in patients with MASLD and coexisting obesity is a disruption of reverse cholesterol transport. There is reduced uptake and excretion of cholesterol from plasma by hepatocytes and adipocytes. The reverse transport of cholesterol is also responsible for HDL; if its levels are low, disruption occurs, so HDL is a marker of MASLD and a risk factor for CVD. However, it is not only HDL levels alone that are important; in the course of MASLD, there is also lipoprotein dysfunction, and antioxidant activity is reduced [73]. The increase in triglycerides depends on a decrease in lipoprotein lipase, which is associated with an increase in angiopoietin-like protein ANGPTL 3 and protein activity ANGPTL 4. Levels of these proteins are increased in patients with obesity, type 2 diabetes, and MASLD. This mechanism causes lipid accumulation in the liver parenchyma and underlies the development of atherosclerosis [74].

### 4.2. Complications Associated with Cardiovascular Disease

#### 4.2.1. MASLD and Hypertension

Due to the frequent co-occurrence of MASLD and obesity, the risk of developing hypertension is very high. Hypertension occurs in about 40% of patients with MASLD [67]. One of the mechanisms explaining this condition is increased activation of the sympathetic nervous system. This is indicated by an increase in neurotransmitters such as norepinephrine and neuropeptide Y [75]. As a result of activation of the sympathetic nervous system, there is increased production of LDL by the liver, which leads to the deposition of atherosclerotic plaques in the vessels, resulting in flow restriction and increased blood pressure. In addition, due to persistent activation of the sympathetic nervous system, there is increased lipolysis in adipocytes, impaired glucose uptake, and insulin resistance, which leads to increased plasma levels of free fatty acids and triglycerides, resulting in visceral obesity [67,75]. In the course of MASLD, there is also a decrease in renal blood flow, glomerular filtration, an increase in sodium retention, and increased activation of the renin–angiotensin–aldosterone system (RAAS)—an increase in activation of the sympathetic nervous system in the liver, a decrease in blood flow, and an increase in hepatic arterial resistance, which activates the renal sympathetic nervous system and, consequently, stimulation of the RAAS [75,76].

#### 4.2.2. MASLD and Structural Abnormalities of the Heart

Studies show that patients with MASLD and coexisting obesity develop concentric left ventricular hypertrophy. Due to increased epicardial adipose tissue, cardiac diastolic function is altered. Numerous available studies have described altered myocardial structure, early left ventricular (LV) systolic, and diastolic dysfunction [67]. Patients with advanced fibrosis (F3–F4) had more epicardial fat than those with milder fibrosis (F0–F2). In addition, other echocardiographic indices, such as posterior diastolic wall thickness, left ventricular mass, relative wall thickness, ejection fraction (EF), and left atrial volume, depended on the severity of hepatic fibrosis [77]. Patients with MASLD exhibit increased LV mass, decreased LV ejection fraction, lower early diastolic relaxation velocity, and increased LV filling pressure, which can lead to heart failure. The mechanisms responsible for these pathologies are the previously mentioned insulin resistance, chronic inflammation, mitochondrial dysfunction, oxidative stress, activation of the sympathetic nervous system, and the RAAS [67].

#### 4.2.3. MASLD and Cardiac Arrhythmias

Many available studies have shown an increased risk of developing cardiac arrhythmias in patients with MASLD, such as atrial fibrillation, ventricular arrhythmias, and QTc interval prolongation. MASLD is associated with cardiac remodeling, which leads to changes in the conduction tissue and induces arrhythmias. In addition, atrial fibrillation (AF), premature ventricular beats, and non-sustained ventricular tachycardia may also occur [78]. Several studies have shown that there is an increased incidence of AF in patients with MASLD, especially with the coexistence of T2DM [68]. This is most likely related to the increased propensity in this group of patients for fat deposition and higher levels of pro-inflammatory, vasoactive, and pro-thrombotic mediators, which can lead to structural and functional disruption of the myocardium and consequently trigger pro-arrhythmogenic effects [79,80].

#### 4.2.4. Ischemic Stroke

The above-described disorders are associated with an increased risk of vascular atherosclerosis, and cardiac arrhythmias can directly lead to ischemic stroke. Patients with MASLD have an increased risk of ischemic stroke [63]. The incidence of stroke in patients with steatohepatic disease in the studies ranged from about 18 to 41% [81,82]. In patients with MASLD, ischemic stroke was more common in middle age [82]. However, biochemical test markers such as alanine aminotransferase (ALT), aspartate aminotransferase (AST), and gamma-glutamyltranspeptidase (GGTP) are not prognostic factors for ischemic stroke [83].

### 4.3. Pharmacological Therapeutic Strategies

Optimal therapeutic management should include both cardiovascular drugs and drugs that improve liver function (Table 1). The drugs used should primarily reduce fibrosis, normalize hepatocyte metabolism, have antioxidant effects, and counteract apoptosis. These mechanisms include the action of vitamin E and ursodeoxycholic acid (UDCA) [72]. Vitamin E reduces steatosis and inflammation of the liver but does not significantly affect the fibrosis process. In addition, vitamin E may increase the risk of hemorrhagic stroke as well as heart failure [72,84]. UDCA improves the secretory function of hepatocytes and inhibits the activity of pro-inflammatory cytokines. The use of UDCA reduces the activity of aminotransferases and reduces hepatic steatosis. However, there is no evidence that it reduces cardiovascular risk [72].

Given the above, specific treatments for MASLD are still lacking. Patients are primarily treated for concomitant diseases. In clinical practice, statins are recommended to reduce the risk of cardiovascular disease complications such as ischemic stroke and myocardial infarction [67]. Statins are inhibitors of 3-hydroxy-3-methylglutaryl coenzyme A reductase (HMGCoA reductase), an enzyme that limits the rate of cholesterol biosynthesis [68]. Their action also involves reducing triglyceride levels in the liver, and they have anti-inflammatory, antiproliferative, anticoagulant, and antioxidant effects [67]. There is no confirmation that fibrates are effective in the treatment of hepatic steatosis. However, combination therapy of ezetimibe and statin has shown a positive therapeutic effect of, among other things, lowering LDL cholesterol levels and improving therapeutic outcomes in patients with MASLD and acute coronary syndrome [63].

Hypoglycemic medications are another point of interest. Examples include sodium-glucose transporter 2 (SGLT2) inhibitors and glucagon-like peptide 1 (GLP-1) receptor agonists approved for the treatment of type 2 diabetes. It appears that these drugs can prevent and delay the progression of MASLD [85]. GLP-1 receptor agonists lower body weight, reduce insulin resistance, and improve plasma lipid levels, all of which can lower the risk of developing CVD [63,85,86]. The ability of SGLT2 inhibitors to exert cardioprotective effects may involve several important pathways beyond their effects on glucose transport, such as reducing oxidative stress, stimulating lipolysis, improving mitochondrial function, suppressing apoptosis, and altering the renin-angiotensin system [85,87]. They have a protective effect on the cardiovascular system and favorable effects on liver function in MASLD, and they may be promising therapeutic options for CVD in patients with MASLD.

Given that one of the mechanisms leading to hypertension in MASLD is activation of the RAAS, the drugs with the greatest potential for treating CVD in MASLD are inhibitors of the RAAS. In addition to the blood pressure-lowering effects of angiotensin II receptor antagonists, studies have demonstrated the ability of these antagonists to improve plasma triglyceride levels, markers of liver damage, and hepatic lipid accumulation [67,88]. Effects on metabolic pathways may be beneficial in patients with MASLD to prevent CVD.

**Table 1:** Mechanisms of selected drug groups having an effect on steatohepatitis associated with metabolic dysfunction (MASLD) and cardiovascular disease (CVD).

Drug/Group of Drugs	Mechanism of Action in MASLD	Mechanism of Action in CVD
Statins [67]	Reduces cholesterol synthesis and liver triglyceride levels	Anti-inflammatory, antiproliferative, anticoagulant, antioxidant
Vitamin E [67,72]	Reduces steatosis and hepatitis	No evidence of reducing cardiovascular risk. May increase the risk of hemorrhagic stroke and heart failure.
Ursodeoxycholic acid [72,89]	Improves hepatocyte secretory function and inhibits pro-inflammatory cytokine activity. Reduces aminotransferase activity and inhibits hepatic steatosis.	No evidence of CVD risk reduction
Hypotensive drug angiotensin II receptor antagonists [89]	Inhibition of the renin–angiotensin–aldosterone system (RAAS), which leads to activation of PPAR-γ, resulting in better control of adipose tissue proliferation and adipokine production	Inhibition of the RAAS lowering blood pressure
Hypoglycemic drugs [90]	Reduces insulin resistance and improves plasma lipid levels	Reduces oxidative stress, stimulates lipolysis, improves mitochondrial function, suppresses apoptosis, and alters the renin-angiotensin system

## 5. Liver Cancer and Metabolic-Related Steatohepatitis

HCC is one of the most common cancers worldwide. HCC is induced in patients with chronic liver disease (CLD), where processes related to damage, inflammation, and regeneration are intertwined [12]. NAFLD, which has recently been given the new name MASLD, is considered the most prevalent liver disease but also one of the main inducers of HCC [91]. MASLD seems to carry a moderate risk of developing HCC; however, due to its prevalence, it has a large proportion of cancer [12,91]. In contrast, a population-based study in the US found that MASLD was the most common risk factor for HCC induction, at 59% [92]. HCC ranks as the fourth most common cause of cancer death worldwide. In addition, it ranks second in loss of life due to cancer. In recent years, an increase in the incidence of HCC has been found. This appears to be due to a combination of aging and population growth, as well as an increase in the prevalence of metabolic syndrome. The highest incidence of HCC and the burden of death from it are found in East Asia [93]. HCC, which is associated with MASLD, is found in the elderly. Importantly, HCC is diagnosed at later stages in these individuals, and they also show lower survival rates compared to patients with HCC associated with viral hepatitis [94]. It also appears that HCC with co-occurring MASLD can develop in the absence of cirrhosis, which in clinical practice potentially contributes to delaying diagnosis [95].

### 5.1. Genetic Factors

In recent years, genes that are associated with MASLD and HCC have been discovered. A nucleotide polymorphism (SNP) in the patatin-like phospholipase domain containing 3 (PNPLA3) gene is responsible for encoding adiponutrin triglyceride lipase. As a result, it is associated with fat deposition in the liver. The PNPLA3 rs738409 [G] risk allele may increase the risk of progression of hepatic steatosis to hepatic inflammation, but importantly, it potentially promotes a 12-fold increased risk of HCC induction. It is worth mentioning that this allele is found in about 40% of the European population [96]. Ertle et al. showed that this allele is closely associated with hepatocancerogenesis in obesity, especially in patients with alcoholic cirrhosis [97]. In addition, there are two genetic variants, which include the c.6718A>T mutation in the apolipoprotein B (ApoB) gene and the c.6718>T mutation in the human telomerase reverse transcriptase (TERT) gene. Both of these variants influence the induction of HCC in the steatotic liver [98].

### 5.2. Gut Microbiota

The gut microbiota, through several different mechanisms, can influence both the development of MASLD and HCC [12]. The mechanisms linking the two disease entities are impaired intestinal barrier permeability and intestinal dysbiosis. It has been shown that high levels of lipopolysaccharide (LPS) are found in patients with CLD and hepatocarcinogenesis. It belongs to the components of the cell wall but is also one of the more potent promoters of inflammation [99]. Studies have shown that intestinal exposure to dextran sulfate sodium (DSS) can contribute to increased LPS levels and systemic inflammation, thereby increasing the risk of HCC in mice. In addition, a high-fat diet in such cases can further increase portal LPS levels and induce inflammation and associated liver fibrosis [100,101]. It seems that it is the process of LPS binding to the Toll-like receptor (TLR) transmembrane receptor that is most important in the induction of HCC. It appears that TLR4 inhibition in mice and rats can reduce inflammation and fibrosis, but also the occurrence of HCC. In turn, TLR4 expression can promote fibrogenesis and the process of hepatocarcinogenesis [102,103]. This can happen at the level of different cells. TLR4 activation in HSCs leads to an increase in epiregulin, which has strong mitogenic effects on hepatocytes. In addition, the LPS–TLR4 axis can reduce hepatocyte apoptosis by utilizing NF-κB. Also, activation of this axis in HCC cells potentially increases epiregulin transition and enhances the invasiveness of these cells [102,103,104]. Intestinal dysbiosis is closely associated with changes in intestinal barrier permeability. Studies have shown changes in the gut microbiota in both CLD and cirrhotic patients. The similarities were characterized by a decrease in beneficial bacteria and an increase in potentially pathogenic bacteria. It seems that these changes are the result of characteristics caused by liver impairment, that is, a decrease in the secretion of intestinal antimicrobial peptides or a reduction in bile secretion [105]. Importantly, not only are changes in the microbiota noted, but also bacterial overgrowth, which is associated with increased amounts of circulating LPS [106]. In addition, LPS acts on liver progenitor cells (HPCs), which it induces to differentiate into myofibroblasts. Through the secretion of TNF-α and IL-6, it can promote inactivation of the p53 tumor suppressor signaling pathway in HPCs and activation of the Ras pathway. As a result, it initiates uncontrolled proliferation and transformation of HPCs, and consequently carcinogenesis [107]. It is also important that the gut microbiota is responsible for the aggregation of liver Natural Killer T cells, and it is these cells, among others, that are responsible for immune mechanisms, which include the production of IFN-γ [108]. Yoshimoto et al. showed that in mice with HCC induced by artificially induced NASH, there was a large increase in Gram-positive bacteria, particularly *Clostridium*. At the same time, they showed an increase in serum deoxycholic acid (DCA), which is a secondary bile acid. It seems that DCA may also be involved in hepatocarcinogenesis [109]. In addition, there are bacterial species, e.g., *Bacteroides fragilis*, that have endotoxic effects. As a result of their action on the Stat3 and Th17 pathways, they can induce the development of HCC [110]. Further research is needed to elucidate the mechanisms involved in the gut–hepatic axis and to uncover new issues with the microbiota that could potentially influence the induction of both MASLD and HCC. Ultimately, the targeting of the gut–liver axis could potentially affect not only the quality of life of patients but also their survival.

### 5.3. Adipose Tissue and Associated Pro-Inflammatory Cytokines

Insulin resistance, which is characterized by chronic low-grade inflammation, promotes macrophage recruitment as well as the induction of pro-inflammatory cytokines. Pro-inflammatory cytokines such as TNF-α and IL-6 are associated with MASLD and the progression of hepatitis to HCC. This appears to happen through mechanisms of action on the signaling pathways IKK and JNK. These pathways are crucial in promoting inflammation induced by excess body weight and liver tumorigenesis [111]. In addition, in obese individuals, high leptin levels are involved in immune regulation and angiogenesis. In turn, leptin binding to the Ob receptor can induce activation of the PI-3K/Akt pathway. Subsequently, mTOR, as one of the Akt effectors, is associated with cell proliferation and growth. It appears that Akt/mTOR activation is confirmed in about 30–40% of HCC patients [112]. In addition, people with obesity have low levels of adiponectin, which may reduce its anti-inflammatory effect [111].

### 5.4. Diet

A high-fat diet can affect the expression of both TNF-α and IL-6 as well as increase NF-κB activation [113]. Zhang et al. showed that a cholesterol-rich diet induces an increased risk of MASLD progression to HCC in mice. This effect appears to arise as a consequence of changes in the gut microbiota and its metabolites [38]. In addition, carbohydrates, especially excess fructose, could potentially activate the transcription factor ChREBP. This factor contributes to the co-occurrence of chronic hyperinsulinemia. Consequently, it induces hepatic lipogenesis de novo. It turns out that it is increased several-fold in obese subjects and MASLD, but importantly, in its process, only saturated fatty acids are produced, and they play a significant role in lipotoxic damage. It appears that fructose may affect the induction of HCC by several mechanisms. These include an increase in lipoperoxidation, which decreases sirtuin-1 expression but may also affect changes in the gut microbiota [114,115].

### 5.5. Other Factors

In addition to the mechanisms linking MASLD to HCC described above, there are other risk factors. These include male gender, older age, and ethnicity [116]. In addition, common risk factors are the presence of obesity and diabetes [117]. Dyson et al. showed that older people with established diabetes or metabolic syndrome had the highest mortality associated with HCC [118]. In turn, Marrero et al. observed that patients with cirrhosis and comorbid obesity were 47 times more at risk for HCC compared to those without liver disease [119].

A summary of factors common to MASDL and other conditions is shown in Table 2.

## 6. Conclusions

In recent years, the prevalence of diseases closely related to MASLD, such as liver cancer, diabetes, obesity, and CVD, has increased significantly. MASLD is part of a major global problem that is now challenging health care. For the treatment of MASLD, most recommendations for “liver detoxification” include a 7–10% weight reduction, in addition to a Mediterranean diet (exclusion of highly processed foods and foods high in fat and fructose), the reduction and preferably elimination of alcohol, and the introduction of physical activity. This last factor should be tailored to the patient’s preferences so as to maintain the conduct of physical activity systematically and in the long term. On the one hand, MASLD contributes to insulin resistance and increased risk of T2DM, and on the other hand, T2DM may lead to the progression of MASLD. In particular, immune-related inflammatory changes, inefficient inhibition of lipolysis, and excess free fatty acids contribute to ectopic lipid deposition and impair insulin signaling in adipose tissue. To counteract unfavorable metabolic changes, all preventive measures should focus on reducing inflammation and oxidative stress in patients. Typical disorders for MASLD, i.e., chronic low-grade inflammation, insulin resistance, or lipid disorders, contribute to cardiovascular disease. In addition, often the final stage of liver disease becomes liver cancer. These risks suggest that MASLD is a multidisciplinary disease, and both cardiologists and gastroenterologists should take extensive preventive measures based on screening high-risk patients.

## Figures and Tables

**Table 2:** Factors common to MASLD and other conditions.

	Obesity [15,16,18,19,21,26,28,32,37]	Type 2 Diabetes [49,50,51,52,53,54,55,56,57,58,59,60,61,62]	Cardiovascular Disease [64,65,66,67,68,69,70,71,72]	Hepatocellular Carcinoma [38,96,97,98,106,110,111,112,113,114]
**Common factors**	Genetic factors: polymorphisms of genes, e.g., patatin-like phospholipase domain containing 3 (PNPLA3) and transmembrane 6 superfamily member 2 (TM6SF2), may increase the production of hepatic triglyceride-rich lipoproteins, which may be associated with higher liver fat content; PPAR-γ is one of the factors that regulates the processes of lipid metabolism and adipocyte differentiation. Therefore, inadequate PPAR-γ function may also be an indirect factor in the association between obesity and MASLD; downregulation of hepatic MBOAT7. Lifestyle: high-fat diet (high-fat diets, in which fat accounts for 45–75% of daily energy needs, have been shown to have an association with the occurrence of hepatic steatosis); high-fructose diet (chronic consumption of large amounts of fructose can predispose to leptin resistance, with indirect effects on weight gain, which in turn can induce the hepatic steatosis cascade and also lead to the induction of excessive oxidative stress and inflammation); sedentary lifestyle (low or no physical activity predisposes to excessive body weight, which, in turn, predisposes to MASLD). Disorders of the intestinal microbiota: increase in *Proteobacteria* and *Firmicutes*; decrease in *Bacteroidetes*.	Chronic inflammation: oxidative stress, inflammatory mediators, lipid peroxidation, and mitochondrial dysfunction-impaired insulin signaling; increase in pro-inflammatory cytokines; insufficient inhibition of lipolysis and excess free fatty acids (FFAs) due to inflammation. Genetic factors: genetic variants (rs58542926 C>T SNP, which encodes the E167K variant of TM6SF2 and GCKR (P446L)). Lifestyle: high dietary intake of fats and carbohydrates can lead to; increased levels of free fatty acids, stimulation of de novo lipogenesis (DNL) leading to reduced secretion of triglycerides (TG) from the liver; increased concentrations of diacylglycerols and ceramides in the liver; hepatic DAG-PKCε-INSR axis	Chronic inflammation: increased production of pro-inflammatory cytokines; increase in the production of CRP protein, oxidized LDL, and plasminogen activator inhibitor-1; insulin resistance by atherogenic dyslipidemia; release of pro-inflammatory factors and vasoactive factors; thrombogenic factors; abnormal lipid profile: high levels triglycerides, low-density lipoprotein cholesterol (LDL-C), reduced HDL-C levels, and high apolipoprotein B (apoB) levels.	Genetic factors: Nucleotide; polymorphism in patatin-like phospholipase domain containing 3 gene allel risk PNPLA3 rs738409; the c.6718A>T mutation in the apolipoprotein B (ApoB) gene and the c.6718>T mutation in the human telomerase reverse transcriptase (TERT) gene. Disorders of the intestinal microbiota: reduced number of beneficial bacteria and an increase in potentially pathogenic bacteria; changes in the permeability of the intestinal barrier; high levels of lipopolysaccharide; Insulin resistance, which promotes macrophage recruitment and induction of pro-inflammatory cytokines; High leptin levels; Low adiponectin levels. Lifestyle: a high-fat diet, which affects the expression of both TNF-α and IL-6 but may also have the effect of increasing NF-κB activation; excess fructose.

## Abbreviations

ACC	acetyl-CoA carboxylase
AF	atrial fibrillation
ALT	alanine aminotransferase
apoB	apolipoprotein B
AST	aspartate aminotransferase
ChREBP	carbohydrate response element-binding protein
CLD	chronic liver disease
CVD	cardiovascular disease
DAG	diacylglycerol
DCA	deoxycholic acid
DNL	de novo lipogenesis
DSS	dextran sulfate sodium
EF	ejection fraction
ER	endoplasmic reticulum
FAS	fatty acid synthesis
FLI	fatty liver index
GGTP	gamma-glutamyltranspeptidase
GLP-1	glucagon-like peptide 1
HCC	hepatocellular carcinoma
HMGCoA	reductase 3-hydroxy-3-methylglutaryl coenzyme A reductase
HOMA-IR	insulin resistance index
HPCs	liver progenitor cells
IHTG	intrahepatic triglycerides
IKK	inhibitor κB kinase
IL-6	interleukin-6
INSR	insulin receptor
IR	insulin resistance
JNK	c-Jun N-terminal kinase
LCD	low-calorie diet
LDL-C	low-density lipoprotein cholesterol
LPS	lipopolysaccharide
LV	left ventricular
MAFLD	metabolic-associated fatty liver disease
	
MASLD	metabolic dysfunction-associated steatohepatitis
NF-κB	nuclear factor kappa-B
PKC	protein kinase C
PKCζ	protein kinase C-ζ
Pklr	l-type pyruvate kinase
PNPLA3	patatin-like phospholipase domain containing 3
PPAR-α	peroxisome proliferator-activated receptor-α
RAAS	renin–angiotensin–aldosterone system
SCD-1	stearoyl CoA-desaturase-1
SGLT2	sodium-glucose transporter 2
SNP	nucleotide polymorphism
SOCS	suppressor of cytokine signaling
Srebp1c	factor binding sterol regulatory element 1
SREBP-1c	sterol regulatory element-binding protein 1c
T2DM	type 2 diabetes
TERT	telomerase reverse transcriptase
TG	triglycerides
TLR	toll-like receptor
TM6SF2	transmembrane 6 superfamily member 2
TNF-α	tumor necrosis factor-α
UDCA	ursodeoxycholic acid
VLKCD	very low-calorie ketogenic diet
WHO	World Health Organization
